# Seasonality of the incidence of bronchiolitis in infants — Brazil, 2016–2022: An interrupted time-series analysis

**DOI:** 10.1590/1984-0462/2025/43/2023203

**Published:** 2024-07-08

**Authors:** Frederico Friedrich, Magali Santos Lumertz, Lucas Montiel Petry, Marina Puerari Pieta, Luana Braga Bittencourt, Bruno Brocker Nunes, Laura de Castro e Garcia, Marcos Otávio Brum Antunes, Marcelo Comerlato Scotta, Renato Tetelbom Stein, Marcus Herbert Jones, Talitha Comaru, Leonardo Araújo Pinto

**Affiliations:** a Pontifícia Universidade Católica do Rio Grande do Sul (PUCRS), Porto Alegre, RS, Brazil.; b Instituto Federal de Education, Ciência e Tecnologia de Farroupilha, Santo Ângelo, RS, Brazil.

**Keywords:** COVID-19, Bronchiolitis, Hospitalization, Infants, COVID-19, Bronquiolite, Hospitalização, Bebês

## Abstract

**Objective:**

To evaluate the seasonality of acute bronchiolitis in Brazil during the 2020–2022 season and compare it with the previous seasons.

**Methods:**

Data from the incidence of hospitalizations due to acute bronchiolitis in infants <1 year of age were obtained from the Department of Informatics of the Brazilian Public Health database for the period between 2016 and 2022. These data were also analyzed by macro-regions of Brazil (North, Northeast, Southeast, South, and Midwest). To describe seasonal and trend characteristics over time, we used the Seasonal Autoregressive Integrated Moving Averages Model.

**Results:**

Compared to the pre-COVID-19 period, the incidence of hospitalizations related to acute bronchiolitis decreased by 97% during non-pharmacological interventions (March 2020 – August 2021) but increased by 95% after non-pharmacological interventions relaxation (September 2021 – December 2022), resulting in a 16% overall increase. During the pre-COVID-19 period, hospitalizations for acute bronchiolitis followed a seasonal pattern, which was disrupted in 2020–2021 but recovered in 2022, with a peak occurring in May, approximately 4% higher than the pre-COVID-19 peak.

**Conclusions:**

This study underscores the significant influence of COVID-19 interventions on acute bronchiolitis hospitalizations in Brazil. The restoration of a seasonal pattern in 2022 highlights the interplay between public health measures and respiratory illness dynamics in young children.

## RESUMO

**Objetivo:**

Avaliar a sazonalidade da bronquiolite aguda no Brasil durante a temporada 2020–2022 e compará-la com a das temporadas anteriores.

**Métodos:**

Os dados de incidência de internações por bronquiolite aguda em lactentes <1 ano de idade foram obtidos do Departamento de Informática da base de dados da Saúde Pública Brasileira para o período entre 2016 e 2022. Esses dados também foram analisados por macrorregiões do Brasil (Norte, Nordeste, Sudeste, Sul e Centro-Oeste). Para descrever características sazonais e de tendência ao longo do tempo, utilizamos o Modelo de Médias Móveis Integradas Autorregressivas Sazonais.

**Resultados:**

Em comparação com o período pré-COVID-19, a incidência de hospitalizações relacionadas com bronquiolite aguda diminuiu 97% durante as intervenções não farmacológicas (março de 2020 – agosto de 2021), mas aumentou 95% após a flexibilização das intervenções não farmacológicas (setembro de 2021 – dezembro de 2022), resultando no aumento geral de 16%. Durante o período pré-COVID-19, as hospitalizações por bronquiolite aguda seguiram um padrão sazonal, que foi interrompido em 2020–2021, mas recuperaram-se em 2022, com um pico ocorrido em maio, aproximadamente 4% superior ao pico pré-COVID-19.

**Conclusões:**

Este estudo ressalta a influência significativa das intervenções contra a COVID-19 nas hospitalizações por bronquiolite aguda no Brasil. A restauração de um padrão sazonal em 2022 sublinha a interação entre as medidas de saúde pública e a dinâmica das doenças respiratórias em crianças pequenas.

## INTRODUCTION

The coronavirus disease 2019 (COVID-19) pandemic led to daycare and school closures and children staying home for several months. The season of the traditional respiratory virus was significantly altered, with notable decreases in the incidence of other seasonal respiratory viral infections.^1^ In 2020, there was a significant reduction in hospitalization for acute bronchiolitis (AB) in children <1 year old in Brazil, on the order of > 70%.^2^

Reports from around the world have revealed up to a 98% reduction in respiratory syncytial virus (RSV) cases during the pandemic.^3,4^ The initial studies came from the Southern Hemisphere countries that were at the beginning of their fall season in March 2020, when the pandemic started.^2,4^

In France, a delayed RSV epidemic in the period that generally corresponds to the end of the epidemic season has been identified.^5^ In Wales, a resurgence of RSV bronchiolitis cases has been observed at a rapid rate that is out of sync with the usual seasonal pattern.^6^ In Western Australia, all-cause hospitalization admissions were observed to initially decline by 35%, then recover to normal levels and then increase by 24% following the cessation of different pandemic period phases compared with expected secular trends.^7^

These dynamics have disrupted traditional AB patterns and assumptions and provide a unique opportunity to learn more about the transmission of respiratory viruses. This seasonal shift and a delayed peak of AB in young children could be encountered in other parts of the world, especially as control measures are relaxed and schools reopen. Further studies are needed to assess the long-term impact of COVID-19 on the incidence of bronchiolitis in infants. This study aims to assess the course of hospitalizations due to acute bronchiolitis in children under one year of age in Brazil during the 2020–2022 season and compare it with previous seasons.

## METHOD

This is a cohort study of hospitalizations for acute bronchiolitis in Brazilian infants under one year of age that uses a retrospective analysis. Data from hospitalizations of AB were obtained from the Department of Informatics of the Brazilian Public Health System (DATASUS) database (http://datasus.saude.gov.br/),^8^ which provides the diagnosis at hospital admission. Further information about DATASUS is described elsewhere.^2,9^

Data on AB hospitalizations were obtained from the DATASUS database for the period of 2016–2022 (month to month) in Brazil. The hospitalization data were obtained from the following links: "Informações de Saúde" (Health Information) (TABNET); "Epidemiológicas e Morbidade"(Epidemiological and Morbidity); "Morbidade Hospitalar" (Hospital Morbidity); "Lista de Morbidade"(Morbidity List). Code J21 of the 10^th^ Revision of the International Classification of Diseases (ICD-10) for AB was used for the age group <1 year and both genders. Exclusion criteria were not considered due to the methodological nature of the study. Also, data from all macro-regions of Brazil were analyzed (North, Northeast, Southeast, South, and Midwest) to account for variations in population density, socioeconomic characteristics, and climate.^10^ On this platform there is no way to access clinical data, only the number of hospitalizations that can be stratified by age (range) and location. Since there is a delay in data entry, data analysis for this article was completed up by the end of December.

Two independent authors reviewed all data to ensure quality. This study does not contain personal or individual data and, therefore, was exempt from evaluation by the Research Ethics Committee. The project received approval from the research system of the Pontifical University of Rio Grande do Sul (SIPESQ, in Portuguese).

To calculate the monthly incidence of hospitalizations in the public health system, we used the following formula: total number of hospitalizations/population number by age (per year and place [Brazil — Brazilian Institute of Geography and Statistics — IBGE]) x 100,000 inhabitants).^11^ The Brazilian National Health Agency provides the percentage of the population that has health insurance per year, and the same percentage was excluded from the denominator, as this population uses other hospital structures and admissions data are not included in DATASUS. These percentages were 25.3% in 2016, 23.1% in 2017, 24.4% in 2018, 24.1% in 2019, 21.2% in 2020, 21.1% in 2021, and 20.8% in 2022 for the population of children <1 year old.^12^

We performed time series analysis using the Seasonal-Autoregressive Integrated Moving Average (SARIMA) model in the R computing environment (version 4.3.1, R Foundation for Statistical Computing, Vienna, Austria. Available at: https://www.R-project.org/),^13^ with packages "Forecast" and "Seas tests" to detect general trends, to perform seasonal decomposition and to find patterns for the theoretical prediction after COVID-19 start. Segmented regression was employed to evaluate the intervention's effect (non-pharmaceutical interventions targeting SARS-CoV-2 on AB hospitalizations) on the study's main outcomes. Monthly data for the last seven seasons were analyzed, with a 12-month seasonality pattern to fit the epidemic curves and make predictions for the last one. A counterfactual was obtained from the SARIMA prediction. Pre-COVID-19 period was from January 2016 to February 2020, and the COVID-19 pandemic period was from March 2020 to December 2022. The COVID-19 period was subdivided into the ‘non-pharmaceutical interventions targeting SARS-CoV-2 (NPIs)’ period (March 2020 to August 2021) and the ‘relaxation of NPIs targeting SARS-CoV-2’ period (September 2021 – December 2022). Seasonality was assessed using the Friedman Test. All graphs were created in an R environment, reflecting the data distribution for graphical representation of temporal trends, randomness, and seasonality. Statistical significance was assumed when p<0.05, considering a 95% confidence interval (CI).

## RESULTS

The study analyzed 252,457 hospital admissions for AB in children <1 year old between 2016 and 2022. Of these admissions, 163,945 were recorded during the pre-COVID-19 period, while the remaining 88,512 occurred during the COVID-19 pandemic.

**Figure 1:**
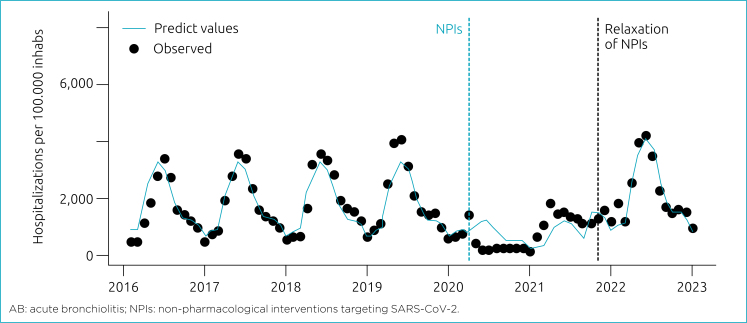
Interrupted time-series of hospitalizations per AB in <1 year.

Compared to the pre-COVID-19 period, there was a 97% reduction (p < 0.01; 95% CI [−0.95 −0.99]) in the incidence of AB hospitalizations during the NPIs (March 2020 – August 2021), a 95% increase (p<0.01; 95%CI 0.92–0.96) after NPIs relaxation (September 2021 – December 2022), and a 16% overall increase (p<0.01; 95%CI 0.14–0.17) when compared to the pre-COVID-19 period (Figure 1).

**Figure 2:**
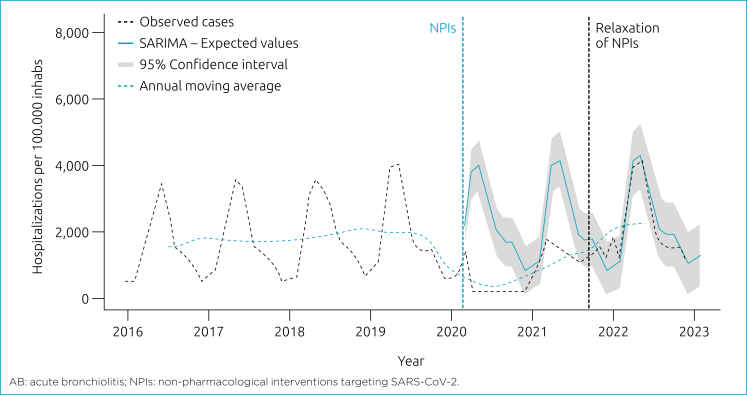
AB seasonality in <1 year old hospitalizations – Brazil.

**Figure 3:**
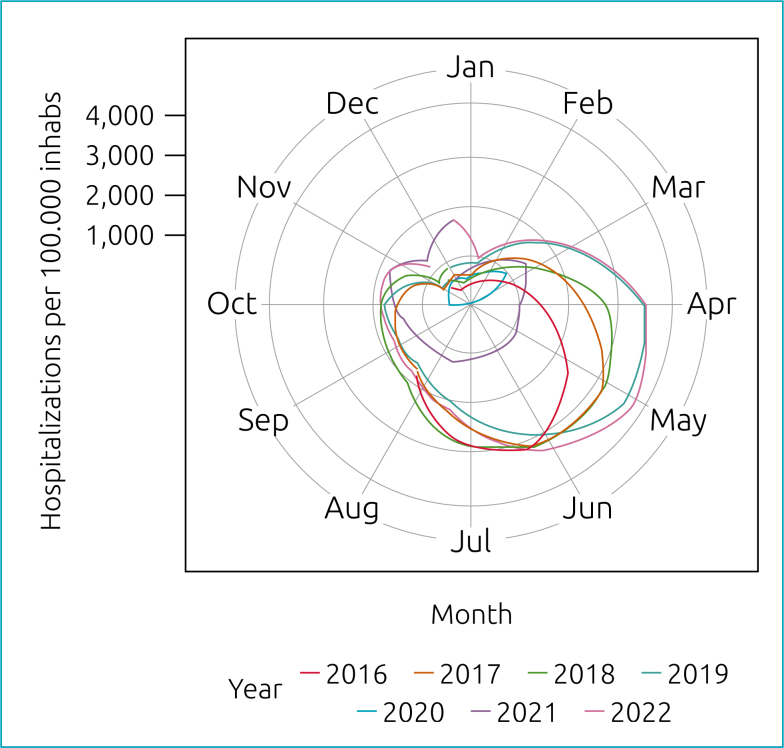
Time-series of hospitalizations per acute bronchiolitis in <1 year.

In terms of seasonal trends, the incidence of hospitalizations for AB followed a seasonal pattern during the pre-COVID-19 period (p<0.01) which was disrupted in 2020–2021 (p=0.201). In 2022, we observed a recovery of the seasonal pattern when compared to the pre-COVID-19 period (p<0.01), with a peak in May, approximately 4% higher than that recorded for 2016–2019 (4,218/100,000 vs. 4,045/100,000) (Figure 2). During the counterfactual (observed vs. expected) period of NPIs, there was an average reduction of 1,400/100,000 hospitalizations. When comparing the expected trend for 2022 with the observed data, we found an increase of 94/100,000 hospitalizations in relation to what was expected (Figure 3).

**Figure 4:**
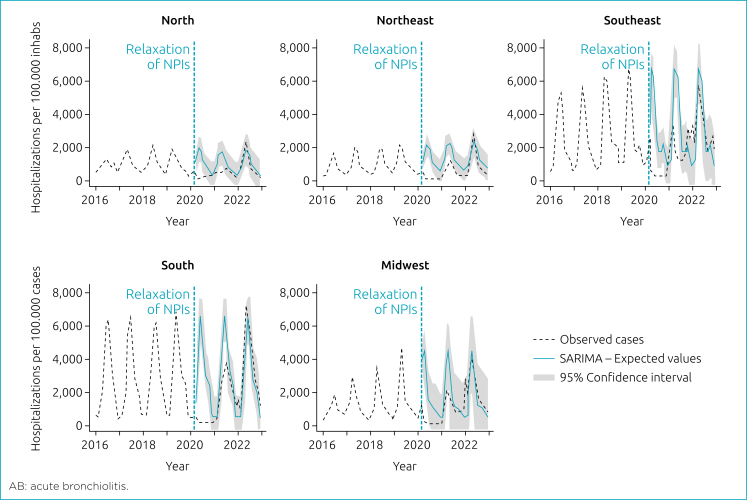
Interrupted time-series of hospitalizations per acute bronchiolitis in <1 year by macro-regions.

When comparing subsets by macro-regions of Brazil (pre-COVID-19 period vs. COVID-19 period), hospitalizations decreased in all regions during the NPIs. The South region experienced the most significant impact, with a reduction from 95 to 3,807/100,000 (observed) vs. 546 to 6,586/100,000 hospitalizations (expected). During the relaxation of NPIs, there was an increase from 761 to 7,262/100,000 (observed) vs. 546 to 6,586/100,000 hospitalizations (expected). The North region showed the least impact during the NPIs period, with a decrease from 323 to 1,964/100,000 (observed) vs. 113 to 766/100,000 hospitalizations (expected). During the relaxation of NPIs, the increase was from 223 to 2,416/100,000 (observed) vs. 275 to 1,890/100,000 hospitalizations (expected) (Figure 4).

## DISCUSSION

During the period spanning 2020–2021, the seasonality of acute bronchiolitis experienced a departure from its typical pattern. However, in 2022, the usual seasonal patterns returned. The relaxation of NPIs seemed to play a significant role in the heightened hospitalizations witnessed during the winter of 2022. This demonstrates the resurgence of typical seasonal patterns of AB.

We examined how the seasonality of AB in Brazilian children changed during the COVID-19 pandemic, compared to previous years. Although it is challenging to quantify the specific impact of pandemic mitigation measures on the spread of common respiratory viruses, it appears that acute respiratory infections in children are reverting to their traditional patterns in Brazil. In the United States, for instance, the pre-pandemic seasons (2017–2020) typically began in October, peaked in December, and concluded in April. However, during the 2020–2021 period, the usual winter RSV epidemic did not occur. Instead, the 2021–2022 season began in May, peaked in July, and ended in January. The 2022–2023 season started in June and peaked in November, which was later than the 2021–2022 season but earlier than the pre-pandemic seasons.^14^ In Spain, a transmission wave with a 7-month delay was observed, starting in early May 2021 and reaching its peak in the first week of July. Throughout this period, schools in Spain remained open from September 2020 until June 2021. In Denmark, where in 2021 RSV presented as a highly unusual out-of-season epidemic, a similar early increase of RSV cases was detected from mid-August. The epidemic reached its peak in November, when the first RSV cases were detected in traditional RSV seasons.^15^ These observations suggest that reopening schools with appropriate safety measures may not have a significant impact on the transmission of SARS-CoV-2, RSV, and other respiratory viruses.^16^

Our findings indicate a resurgence of AB hospitalizations in the year 2022, with numbers slightly higher than in previous seasons. This epidemiological shift was previously observed in the southern hemisphere.^17^ In Australia^18^ and New Zealand,^19^ following a winter season in 2020 with almost nonexistent RSV, an unexpected resurgence of the virus occurred during the summer, resulting in an even larger outbreak compared to previous epidemic seasons.

We also observed an interseasonal spread, which has been relevant in other countries,^20^ in our study. These findings underscore the significance of maintaining robust epidemiological surveillance systems to monitor the trends in bronchiolitis. Additionally, these data provide further confirmation of the effectiveness of universal prophylaxis in preventing hospitalization of infants who do not currently meet the recommended criteria for palivizumab treatment.^21^ It also highlights the potential benefits of implementing RSV vaccines and new monoclonal antibodies.^22^

One limitation of this study is that we used information regarding hospital admissions from a third-party database. To obtain this data, we collected information for each month with a two-month delay. Based on our previous experiences,^2,9^ this period was sufficient for DATASUS to present the final numbers or even very approximate values, as the data are included based on hospitalization authorization forms (Hospital Admission Authorization — AIH) in Brazil. Another limitation of the study was its inability to include cases of acute bronchiolitis and hospitalizations in supplementary healthcare, which represented 21 to 25% of the population during the years of the study.

Furthermore, the data have a population-based nature, making Brazil likely to possess more robust national and regional epidemiological data than any other country in the Southern Hemisphere. This strengthens the findings and presents an advantage over other recent studies. Despite these limitations, we believe that the results genuinely reflect the current moment, showing an increase and return of seasonality in the number of hospitalizations for AB in Brazil.

While the 2022 season calendar suggests that seasonal patterns are returning to those seen in pre-pandemic years, clinicians should be aware that off-season AB hospitalizations may continue. In addition, accurate surveillance data can guide decisions about administering immunoprophylaxis, such as new RSV vaccines and monoclonal antibodies, to high-risk populations. Identifying periods of increased viral circulation can help optimize the timing and targeting of preventive interventions, reducing the burden of serious respiratory illness in vulnerable individuals, particularly infants and young children.

## Data Availability

The database that originated the article is available with the corresponding author.
